# Calcium-dependent ultrasound stimulation of secretory events from pancreatic beta cells

**DOI:** 10.1186/s40349-017-0108-9

**Published:** 2017-12-05

**Authors:** Ivan Suarez Castellanos, Tania Singh, Bogdan Balteanu, Diti Chatterjee Bhowmick, Aleksandar Jeremic, Vesna Zderic

**Affiliations:** 1 Department of Biomedical Engineering, The George Washington University, 800 22nd St. NW rm 5290, Washington, District of Columbia 20052 USA; 2 Department of Biological Sciences, The George Washington University, Washington, District of Columbia USA

## Abstract

**Background:**

Our previous studies have indicated that ultrasound can stimulate the release of insulin from pancreatic beta cells, providing a potential novel treatment for type 2 diabetes. The purpose of this study was to explore the temporal dynamics and Ca^2+^-dependency of ultrasound-stimulated secretory events from dopamine-loaded pancreatic beta cells in an in vitro setup.

**Methods:**

Carbon fiber amperometry was used to detect secretion from INS-1832/13 beta cells in real time. The levels of released insulin were also measured in response to ultrasound treatment using insulin-specific ELISA kit. Beta cells were exposed to continuous wave 800 kHz ultrasound at intensities of 0.1 W/cm^2^, 0.5 W/cm^2^ and 1 W/cm^2^ for several seconds. Cell viability tests were done with trypan blue dye exclusion test and MTT analysis.

**Results:**

Carbon fiber amperometry experiments showed that application of 800 kHz ultrasound at intensities of 0.5 and 1 W/cm^2^ was capable of stimulating secretory events for durations lasting as long as the duration of the stimulus. Furthermore, the amplitude of the detected peaks was reduced by 64% (*p* < 0.01) when extracellular Ca^2+^ was chelated with 10 mM EGTA in cells exposed to ultrasound intensity of 0.5 W/cm^2^. Measurements of released insulin in response to ultrasound stimulation showed complete inhibition of insulin secretion by chelating extracellular Ca^2+^ with 10 mM EGTA (*p* < 0.01). Viability studies showed that 800 kHz, 0.5 W/cm^2^ ultrasound did not cause any significant effects on viability and metabolic activity in cells exposed to ultrasound as compared to sham-treated cells.

**Conclusions:**

Our results demonstrated that application of ultrasound was capable of stimulating the release of insulin from pancreatic beta cells in a safe, controlled and Ca^2+^-dependent manner.

## Background

Type 2 diabetes mellitus is a complex metabolic disease that has reached epidemic proportions in the United States, affecting approximately 27.9 million people as of 2014, with an additional 1.4 million people being diagnosed every year [1–3]. Type 2 diabetes patients often develop multiple complications ranging from diabetic retinopathy – the most frequent cause of new cases of blindness among adults from 20 to 74 years old – to neuropathy which results in numbness, tingling or pain at varying levels of severity [4, 5]. Nephropathy and end-stage renal disease are also common complications of type 2 diabetes, especially among long-term sufferers [6]. Patients with type 2 diabetes also experience increased incidence and poorer outcomes from myocardial infarctions and strokes [7, 8].

The accepted model of stimulus-induced secretion in pancreatic beta cells involves a sequence of events including the closure of ATP-sensitive potassium channels, membrane depolarization, an influx of Ca^2+^ leading to a rise in the intracellular calcium concentration, and exocytosis of insulin [9, 10]. Type 2 diabetes results from the interplay of systemic insulin resistance of peripheral tissues, insufficient insulin secretion from pancreatic beta cells, and insufficient beta cell mass due to genetic and environmental factors [11–14]. As the disease progresses, significant beta cell mass either undergoes apoptosis or becomes “glucose-blind”, wherein beta cells still produce and store insulin but glucose does not mobilize intracellular calcium leading to the lack of insulin release [12, 15]. Although the impairment of insulin action – the interaction between insulin and peripheral tissues – is found in almost all type 2 diabetes patients, it is the impairment of insulin secretion that accounts for the development of hyperglycemia and the progression of the disease [16]. According to the United Kingdom Prospective Diabetes Study (UKPDS), beta cell function is already reduced by up to 50% at the time that diabetes is diagnosed, and continues to decline progressively, regardless of treatment, in subsequent years [17]. This finding demonstrates that substantial defects in beta cell function develop much earlier than the diagnosis of hyperglycemia and that the progression of the disease is mainly driven by the decline of insulin secretion. Even in the presence of insulin resistance, near-normal glucose tolerance in a patient can be achieved so long as there is sufficient insulin secretion to meet the elevated requirements necessitated by insulin resistance. Thus, the disease progresses cyclically in that the initial defect in secretion contributes to progressive metabolic deterioration, which in turn leads to a further decline in beta cell mass and function [16]. However, islet dysfunction is not always irreversible. Any intervention that can improve blood glucose levels can improve beta cell function to an extent [18]. However, current pharmacological treatment courses of type 2 diabetes are very complex and include many side effects which can result in further complications [19–23].

We have previously shown that the application of ultrasound to pancreatic beta cells induces up to four fold increases in release of insulin as compared to control, while retaining cell viability [24]. Further, previous published studies demonstrated that ultrasound application can stimulate calcium transients in cells [25]. In view of this, we hypothesize that the observed increase in insulin secretion is the result of ultrasound-stimulated calcium influx and the subsequent triggering of insulin vesicle exocytosis [26–30]. This study seeks to further elucidate the mechanism by which ultrasound induces secretion and the role that calcium plays in that process. To the best of our knowledge, this is the first study aimed at elucidating the potential mechanisms of ultrasound stimulation of pancreatic beta cell secretion.

## Methods

### Cell preparation

One day before treatment, INS-1832/13 beta cells (gift in kind from C. Newgard’s lab), glucose-responsive insulin secreting insulinoma cell line, were trypsinated and resuspended in fresh RPMI-1640 tissue culture medium. The number of cells in the suspension was determined using a trypan blue dye and a TC20 automatic cell counter (Bio-Rad Laboratories, Inc. Hercules, CA, USA). Briefly, 10 μL of the cell suspension was acquired and mixed with 10 μL of 0.5% trypan blue solution (Bio-Rad Laboratories, Inc. Hercules, CA, USA). Ten μL of the mix were acquired and placed on a dual chamber cell counting slide (Bio-Rad Laboratories, Inc. Hercules, CA, USA). The cell counting slide was then loaded in a TC20 automatic cell counter and the final count was used to dilute and adjust the cell concentration to 3.5–4 × 10^5^ cells/mL. Two milliliters of the INS-1832/13 cell suspensions were plated on 35 mm diameter polystyrene Petri dishes (Corning Life Sciences, Corning, NY, USA) (7–8 × 10^5^ cells in total) and incubated for 16–20 h in media. After this initial incubation phase, the culture medium in each well was changed with medium supplemented with 0.1 mM of dopamine hydrochloride and 0.1 mM of L-DOPA ((3,4-Dihyrdoxy-L-phenylalanine) and further incubated for 4–6 h to let the neurotransmitters load into the secretory vesicles. These neurotransmitters were used as a surrogate for insulin release and detection as they are readily oxidizable by carbon fiber electrodes. Before experimentation the media was removed, and 2.55 mL of modified Krebs bicarbonate solution (KBS), a salt solution used to emulate physiological conditions, was added to each well (138 mM NaCl, 5.4 mM KCl, 1 mM MgCl_2_, 2.6 mM CaCl_2_, 5 mM NaHCO_3_, 10 mM HEPES, 0.1% BSA and pH of 7.4).

### Carbon fiber amperometry

In this study, carbon fiber amperometry was used to study ultrasound-induced insulin secretion dynamics with high temporal resolution**.** Carbon fiber amperometry is a technique in which a carbon fiber electrode is held at a potential higher than the oxidizing potential of the secreted molecule [31, 32]. When the secreted molecule comes into contact with the electrode, a reduction-oxidation reaction occurs on the surface of the electrode, producing a detectable current [32]. Since the method measures only exocytosis, it can detect infrequent exocytotic events without being disturbed by concurrent endocytosis [33]. However, because insulin is not readily oxidizable, it is necessary to artificially introduce molecules into the insulin-containing vesicles that will be released concomitant with insulin and oxidize when they come in contact with the electrode [34–36]. The accumulation of neurotransmitters, such as dopamine and serotonin, within the neurosecretory vesicles of pancreatic beta cells has long been established [37, 38] Uptake of L-DOPA, which is internally converted to dopamine, has been shown specifically in RIN-m5f and INS-1 cells [39–41]. When a stimulus is applied, these neurotransmitters are co-released with insulin [39].

**Fig. 1:**
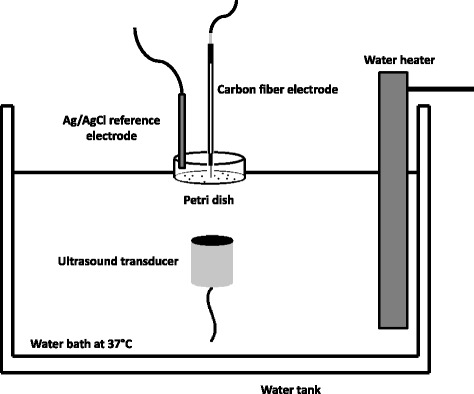
Experimental setup

Petri dishes were carefully placed at the surface of a temperature-controlled water bath maintained at 37 °C using a micropositioner as shown in Fig. 1. A circular planar ultrasound transducer with an active diameter of 1.5 cm and center frequency of 800 kHz (Sonic Concepts, Inc. Bothell, WA, USA) was immersed into the water and placed 3 cm away from the bottom of the Petri dish (distance corresponding to the near-field to far-field transition distance of the transducer) using a 3-D micropositioning system with 0.025 mm resolution and the transducer’s active area directed upwards towards the center of the dish (see Fig. 1). Ultrasound waveforms were generated using an Agilent 33220A function generator (Agilent Technologies, Santa Clara, CA, USA) and were amplified (50 dB gain) using a 150A100B RF amplifier (Amplifier Research, Souderton, PA, USA).The tip of the carbon fiber electrode was carefully immersed into the solution with a micropositioner and placed near the bottom of the well where the monolayer of cells was located. An Ag/AgCl reference electrode was also immersed into the solution near the side wall of the Petri dish where we expect little to no ultrasound exposure. We estimated, using finite-element modelling of the experimental setup, that the ultrasound beam will stimulate approximately 26.6% of the plated cells around the center of the plate and the cells located near the edges of the dish will remain largely unstimulated. Oxidation currents detected with the carbon fiber electrode were recorded with a MicroC Potentiostat (World Precision Instruments, Inc., Sarasota, FL, USA), and sampled at a rate of 100 kHz with a data acquisition device (USB-6003, National Instruments, Austin, TX) and further processed with LabView (Texas Instruments, Dallas, TX, USA). The data was filtered with a digital 4th order Butterworth low-pass filter with a cutoff frequency of 40 Hz. Continuous-wave ultrasound was applied at intensities of 0.1 W/cm^2^, 0.5 W/cm^2^ and 1 W/cm^2^ for durations of 5, 10 and 15 s at *t* = 1 min, *t* = 2 min and *t* = 3 min, respectively. Data was recorded for 4 min per treatment.

Samples were treated in pairs. Comparative pairs were dopamine-loaded cells treated with 800 kHz ultrasound at intensity 0.5 W/cm^2^ (*n* = 6) and unloaded cells treated with 800 kHz ultrasound at intensity 0.5 W/cm^2^ (*n* = 6); and dopamine-loaded cells treated with 800 kHz ultrasound at intensity 0.5 W/cm^2^ (*n* = 6) with dopamine-loaded cells treated at intensity 0.1 W/cm^2^ (*n* = 5). In a separate set of experiments, 10 mM of EGTA (Ethylene glycol-bis(beta-aminoethyl ether)-N,N,N′,N′-tetraacetic acid tetrasodium salt), a known Ca^2+^ chelator, was added to the KBS. Detected currents from ultrasound-treated cells at intensities of 0.5 W/cm^2^ and 1 W/cm^2^ with and without chelation of Ca^2+^ (*n* = 6) were compared as to highlight the role and impact of Ca^2+^ in ultrasound-induced secretory events. Samples were also treated in pairs. All paired samples were treated on the same day. The data was analyzed by calculating the area under the curve during each stimuli/response timeframe (e.g. 5 s at *t* = 1 min, 10 s at *t* = 2 min and 15 s at *t* = 3 min).

### ELISA studies

Experiments demonstrating that amperometric detection of released dopamine was accompanied by release of insulin were performed by measuring levels of released insulin from beta cells treated with ultrasound as compared to sham-treated cells. Measurements of insulin release were performed on samples treated with 800 kHz ultrasound at intensity of 0.5 W/cm^2^ applied for 15 s (*n* = 4), samples treated with 800 kHz ultrasound at intensity of 0.5 W/cm^2^ applied for 5 min (*n* = 4), and sham-treated cells (*n* = 4). Briefly, 200 μL aliquots of KBS from all dishes were collected at *t* = 0 min (before the start of ultrasound treatment) and immediately after the end of treatment (15 s or 5 min depending on the duration of the ultrasound exposure). Insulin concentration in collected samples was determined using an ELISA Insulin Kit (Millipore Corporation, Billerica, MA, USA) and a SpectraMax M5 Spectrometer (Molecular Devices, Sunnyvale, CA, USA). Briefly, after preparing all samples according to instructions from the colorimetric ELISA kit, absorbance of each sample at 590 nm were measured using the spectrometer and corrected for their respective background signal at 450 nm. Insulin concentration within each sample was determined by the linear relationship between absorbance and insulin concentration (for concentrations ranging from 0.2 ng/mL to 10 ng/mL) as determined by calibration of the ELISA kit. As such, collected samples were diluted until their measured insulin concentrations fell within the linear range of the ELISA assay (0.2 ng/ml to 10 ng/ml). The final insulin concentration was determined by multiplying the measured concentration by its respective dilution factor. Insulin release at the end of treatment was quantified by taking the difference between the measured extracellular insulin concentrations at *t* = 15 s or *t* = 5 min (depending on the duration of treatment) and their respective control value at *t* = 0 min. We expected to measure positive differences for enhanced insulin release, differences close to zero for no effect on insulin release and negative differences for decreased extracellular insulin content. Additionally, measurements of insulin release with and without 10 mM EGTA were performed on samples treated with 800 kHz ultrasound at intensity of 0.5 W/cm^2^ applied for 5, 10 and 15 s at *t* = 1 min, *t* = 2, min and *t* = 3 min (*n* = 6 per group). The aim of these experiments was to further highlight the role of Ca^2+^ in the process of ultrasound-stimulated insulin release.

### Cell viability

Cell viability 24 h after treatment was characterized using 3-(4,5-dimethylthiazol-2-yl)-2, 5-diphenyltetrazolium bromide (MTT) reduction cytotoxic assay and trypan-blue exclusion method as described previously [42]. In these experiments, Petri dishes were coated with fibronectin as to reduce any potential cell detachment in response to ultrasound treatment.

Immediately after 15 s of 800 kHz, 0.5 W/cm^2^ ultrasound treatment, the cells in all treated wells were chemically detached through trypsination, counted using a trypan blue dye and a TC20 automatic cell counter (Bio-Rad Laboratories, Inc. Hercules, CA, USA), and re-plated in a 12-well tissue culture plate at a cell density of ~ 3–4 × 10^5^ cells per well. The plate was incubated for 24 h before MTT cytotoxicity analysis. Formazan crystals, a product of MTT reduction, are indicators of the pyridine nucleotide redox state of the cell and thus an indicator of cell metabolic activity. The day following ultrasound treatment, the cell medium in all samples was replaced with 1 mL of KBS containing 1 mg/mL of MTT solution and incubated for 3 h at 37 °C. Converted formazan crystals in each sample were dissolved in isopropanol/HCl solution and quantified by measuring the absorbance of the resulting formazan dye at 570 nm using a SpectraMax M5 Spectrometer (Molecular Devices, Sunnyvale, CA, USA). Cells in samples exhibiting higher metabolic activity will result in higher measured levels of absorbance as compared to samples containing apoptotic or necrotic cells. The measured absorbance in each sample was normalized to its respective cell count. MTT cytotoxicity assay results from ultrasound-treated cells (*n* = 6) were compared to results obtained from sham-treated cells (*n* = 6).

### Modeling studies

The experimental setup shown in Fig. 1 was modeled using PZFlex modeling software (Weidlinger Associates, Mountain View, CA, USA). The purpose of these simulations was to establish a range of pressures to which the cells were exposed to as result of the formation of standing waves or interference patterns within the Petri dish. Further, simulations provided pressure maps at very high spatial resolution, therefore better characterizing the acoustic field affecting the cells. Simulation parameters in the PZFlex model were established as previously reported [43]. Material properties, parameters and dimensions were obtained from our measurements, manufacturers’ data and published data. Material properties used in our simulations for water and polystyrene Petri dishes are shown in Table 1. The grid size was set to one fifteenth of the exposure wavelength to ensure proper spatial resolution as recommended by the PZFlex software manufacturer [44]. An 800 kHz pressure sine-wave of 100 cycles and 187 kPa peak-negative pressure was applied to the model. Simulated pressure calculations were compared to point measurements obtained experimentally with an acoustic hydrophone (HGL-0085, Onda Corporation, Sunnyvale, CA) placed directly into the solution at bottom of the Petri dish.

**Table 1:** Acoustic properties of materials used in simulations compiled from manufacturer’s data and literature

Material	Density (kg/m^3^)	Bulk modulus (MPa)	Shear modulus (MPa)
Water [73]	1000	2200	0
Polystyrene [74]	1052	3592	1270

## Results

**Fig. 2:**
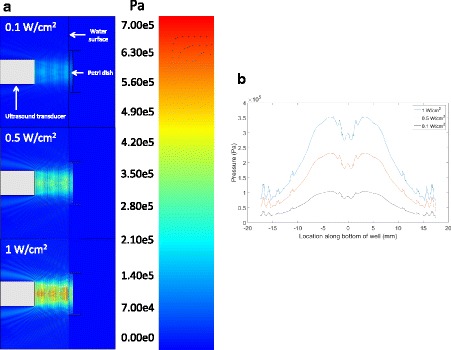
**a** Pressure maps of experimental setup (side view) generated using PZFlex modeling software for 800 kHz ultrasound applied at intensities of 0.1 W/cm^2^, 0.5 W/cm^2^ and 1 W/cm^2^. **b** Pressure profiles at the location of the cell monolayer at the bottom of the Petri dish for 800 kHz ultrasound applied at intensities of 0.1 W/cm^2^, 0.5 W/cm^2^ and 1 W/cm^2^

Pressure maps of our experimental setup were generated for the different ultrasound frequencies used experimentally (Fig. 2a). The pressure profiles at the bottom of the dish (where the beta cell monolayer is located) for intensities of 0.1 W/cm^2^, 0.5 W/cm^2^ and 1 W/cm^2^ are displayed in Fig. 2b. According to these simulations, the cell monolayer was exposed to maximum peak rarefactional pressures in the range of 95 ± 7 kPa (mean ± STD), 211 ± 17 kPa (mean ± STD) and 322 ± 25 kPa (mean ± STD) for intensities of 0.1 W/cm^2^, 0.5 W/cm^2^ and 1 W/cm^2^, respectively. All three profiles were similar in shape, having local pressure maxima at distances of 3.55 mm away from the center of the dish (see Fig. 2b). Comparison of simulated pressure calculations with measurements obtained experimentally with an acoustic hydrophone resulted in differences no higher than 20%.

**Fig. 3:**
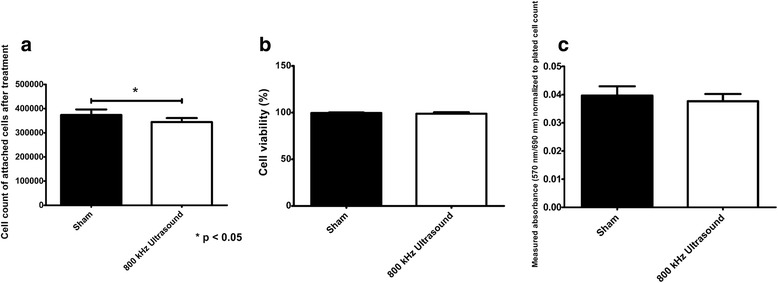
**a** Cell count of trypsinated cells after 800 kHz ultrasound treatment at intensity of 0.5 W/cm^2^ (*n* = 5, mean ± STD) and sham (*n* = 5, mean ± STD) treatment. **b** Cell viability immediately after 800 kHz ultrasound treatment at intensity of 0.5 W/cm^2^ (*n* = 5, mean ± STD) and sham treatment (*n* = 5, mean ± STD) as measured using trypan blue dye exclusion test (Petri dish setup). **c** MTT cytotoxicity assay comparing 800 kHz, 1 W/cm^2^ ultrasound-treated group (*n* = 5, mean ± STD) and sham-treated group (*n* = 5, mean ± STD)

To demonstrate that ultrasound treatment of pancreatic beta cells does not cause significant effects on cell viability, we performed trypan blue dye exclusion test and MTT cytotoxicity assay to assess cell integrity and function of cells stimulated with ultrasound. Fig. 3a shows the cell count of cells that remained attached to the Petri dish after ultrasound treatment (*n* = 5) and sham treatment (*n* = 5) as counted with an automatic cell counter. The cell count for the ultrasound-treated group was 3.44 ± 0.16 × 10^5^ cells/mL (*n* = 5, mean ± STD) compared to a cell count of 3.74 ± 0.22 × 10^5^ cells/mL (*n* = 5, mean ± STD) in the sham-treated group. As such, there was approximately a 9% reduction in the cell count of the ultrasound-treated samples compared to the sham group (*p* < 0.05). Cell viability measurements, obtained using trypan blue dye exclusion test and performed immediately after ending ultrasound treatment, are displayed in Fig. 3b. No statistical significance was achieved (*p* < 0.05) between cell viability in the ultrasound-treated group (*n* = 5, 98.8 ± 1.64%) and the sham group (*n* = 5, 98.8 ± 1.64%). MTT cytotoxicity assay results performed on ultrasound-treated and sham-treated groups 24 h after treatment are shown in Fig. 3c. No statistical significance was obtained in measurements of absorbance at 570 nm/690 nm (*p* < 0.05) from ultrasound-treated cells (*n* = 5, 0.038 ± 0.0026) and absorbance from sham-treated cells (*n* = 5, 0.04 ± 0.0033), thus showing similar levels of metabolic activity in both groups.

**Fig. 4:**
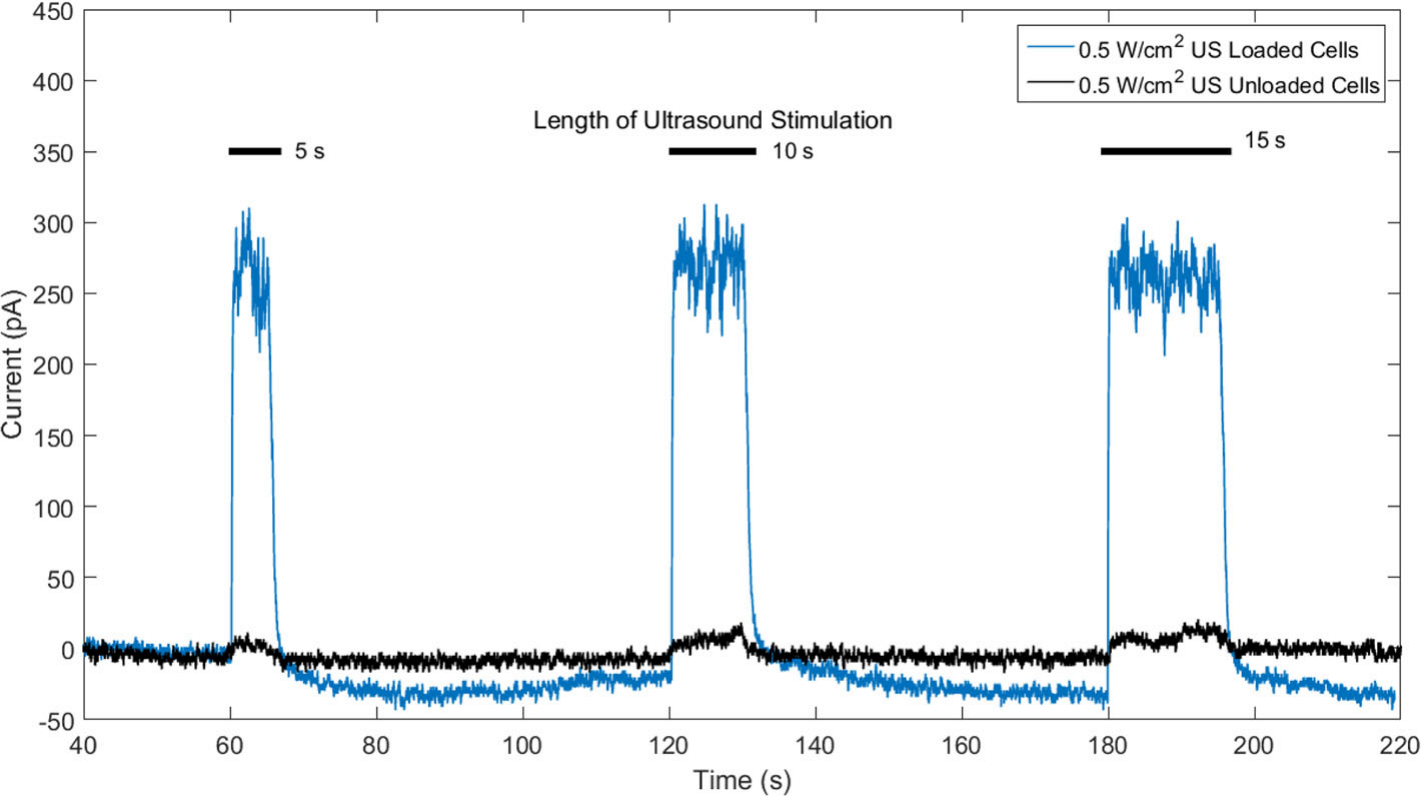
Amperometric peaks detected after 800 kHz ultrasound (US) stimulation with intensity of 0.5 W/cm^2^ applied to dopamine-loaded cells and non-loaded cells

The carbon fiber amperometry experiments were aimed at demonstrating that amperometric peaks were detected from dopamine-loaded cells stimulated with ultrasound as compared to unloaded cells. Figure 4 shows an example of the current detected by the carbon fiber electrode in response to dopamine release from ultrasound-stimulated beta cells (*n* = 6) as compared to the signal recorded from unloaded beta cells exposed to ultrasound using the same parameters (*n* = 6). As expected, peaks from unloaded cells (black curve) are almost negligible compared to peaks detected from loaded beta cells (blue curve). Furthermore, the duration of the amperometry signal resulting from dopamine detection appeared to last as long as the duration of ultrasound stimulation. Table 2 contains the charge (mean ± STD, in pAs – picoAmperes·seconds) measured after each ultrasound stimulating pulse applied to samples containing dopamine-loaded beta cells and those containing unloaded cells. Measurements of detected currents in ultrasound-treated cells loaded with dopamine were significantly higher than currents detected from unloaded cells exposed to ultrasound treatment (Table 2 **,** *n* = 6, *p* < 0.001), demonstrating detection of dopamine-release resulting from ultrasound stimulation.

**Table 2:** Total charge of amperometric peaks for ultrasound-treated cells loaded and unloaded with dopamine (*n* = 6, mean ± STD)

			Total charge (pAs)		
Frequency (kHz)	Intensity (W/cm^2^)	Dopamine loaded?	1 min (5 s)	2 min (10 s)	3 min (15 s)
800	0.5	YES	2700 ± 967	5319 ± 2101	7319 ± 2379
800	0.5	NO	55 ± 21	62 ± 30	92 ± 37

**Fig. 5:**
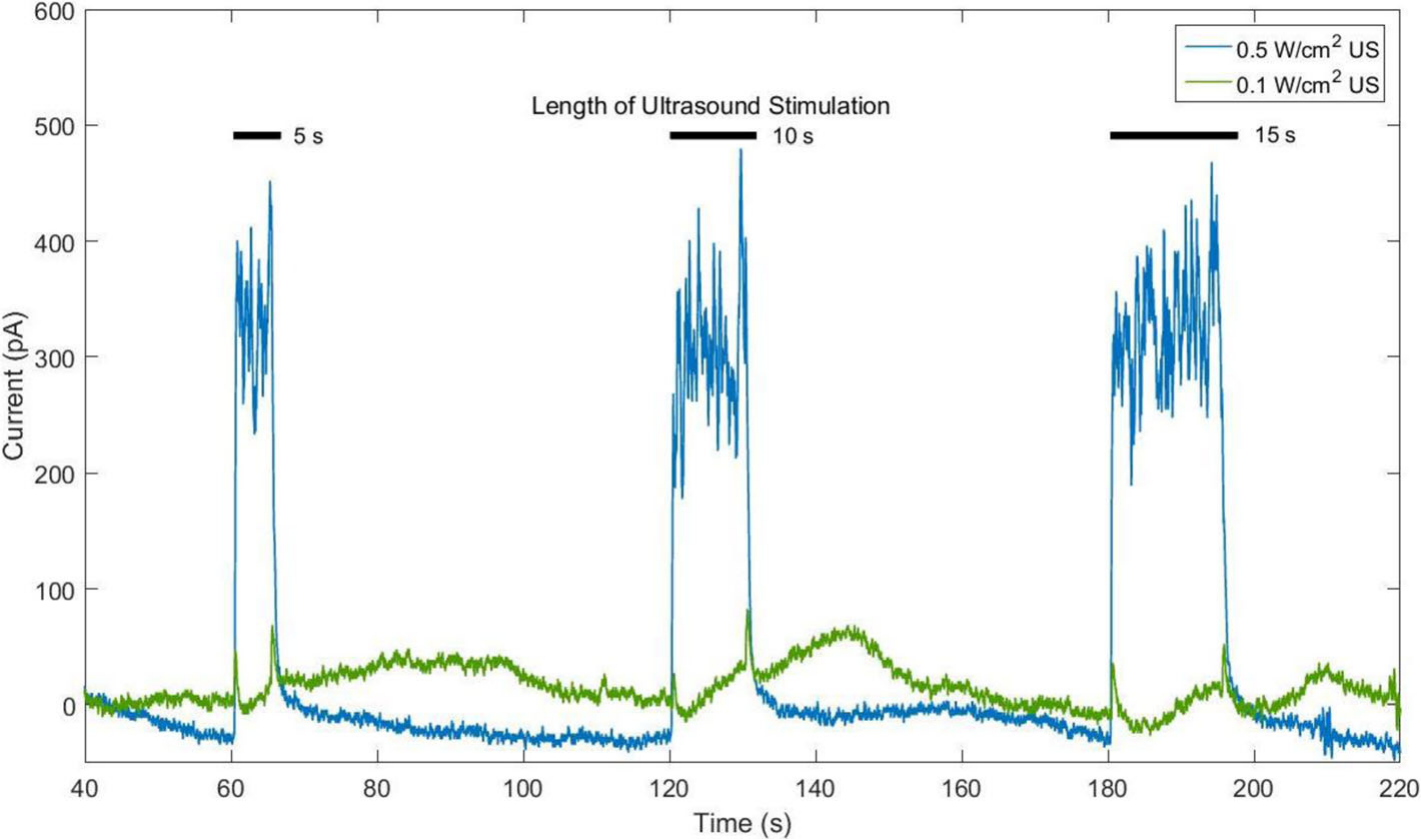
Amperometric peaks detected after 800 kHz ultrasound (US) stimulation at intensities of 0.5 W/cm^2^ (blue curve) and 0.1 W/cm^2^ (red curve)

The follow-up studies were also performed to determine the effect of stimulating beta cells with two different ultrasound intensities. Figure 5 shows an example of currents detected as a result of ultrasound stimulation applied at a frequency of 800 kHz and intensities of 0.5 W/cm^2^ (*n* = 6) and 0.1 W/cm^2^ (*n* = 6). Table 3 contains the total charge (mean ± STD) measured from beta cells exposed to 800 kHz ultrasound pulses of 5, 10, and 15 s at intensities of 0.5 W/cm^2^ and 0.1 W/cm^2^. Detected currents resulting from ultrasound stimulation at intensity of 0.5 W/cm^2^ were significantly higher (approximately 8-fold higher) than currents detected from stimulation at intensity of 0.1 W/cm^2^ (Table 3 **,** *n* = 6, *p* < 0.001).

**Table 3:** Total charge of amperometric peaks for cells treated with 800 kHz ultrasound at intensities of 0.5 W/cm^2^ and 0.1 W/cm^2^ (*n* = 6, mean ± STD)

		Total charge (pAs)		
Frequency (kHz)	Intensity (W/cm^2^)	1 min (5 s)	2 min (10 s)	3 min (15 s)
800	0.5	1163 ± 403	2242 ± 541	3587 ± 1082
800	0.1	158 ± 101	160 ± 41	425 ± 22

**Fig. 6:**
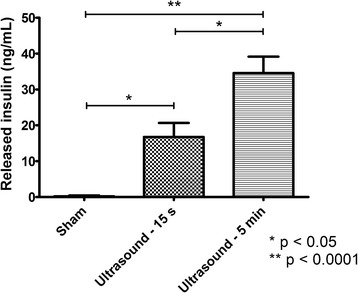
Released insulin from 800 kHz ultrasound-treated cells for treatment durations of 15 s and 5 min and sham-treated cells (*n* = 4, mean ± SEM)

The purpose of the ELISA studies was to demonstrate that detection of amperometric peaks in response to ultrasound stimulation in this experimental setup was accompanied by the release of insulin. Figure 6 shows the measured levels of released insulin from beta cells exposed to 800 kHz ultrasound at intensity of 0.5 W/cm^2^ for durations of 15 s (*n* = 4) and 5 min (*n* = 4) compared to levels from sham-treated cells (*n* = 4). No released insulin was detected in sham-treated groups. Cells treated with 800 kHz continuous ultrasound at intensity of 0.5 W/cm^2^ for duration of 15 s released approximately 16.8 ng/mL of insulin (*n* = 4, *p* < 0.05) while cells exposed to 5 min using the same parameters released approximately 34.6 ng/mL of insulin (*n* = 4, *p* < 0.001). Both 15 s and 5 min ultrasound exposure stimulated significant insulin release from beta cells as compared to sham-treated cells (*n* = 4, *p* < 0.05).

**Fig. 7:**
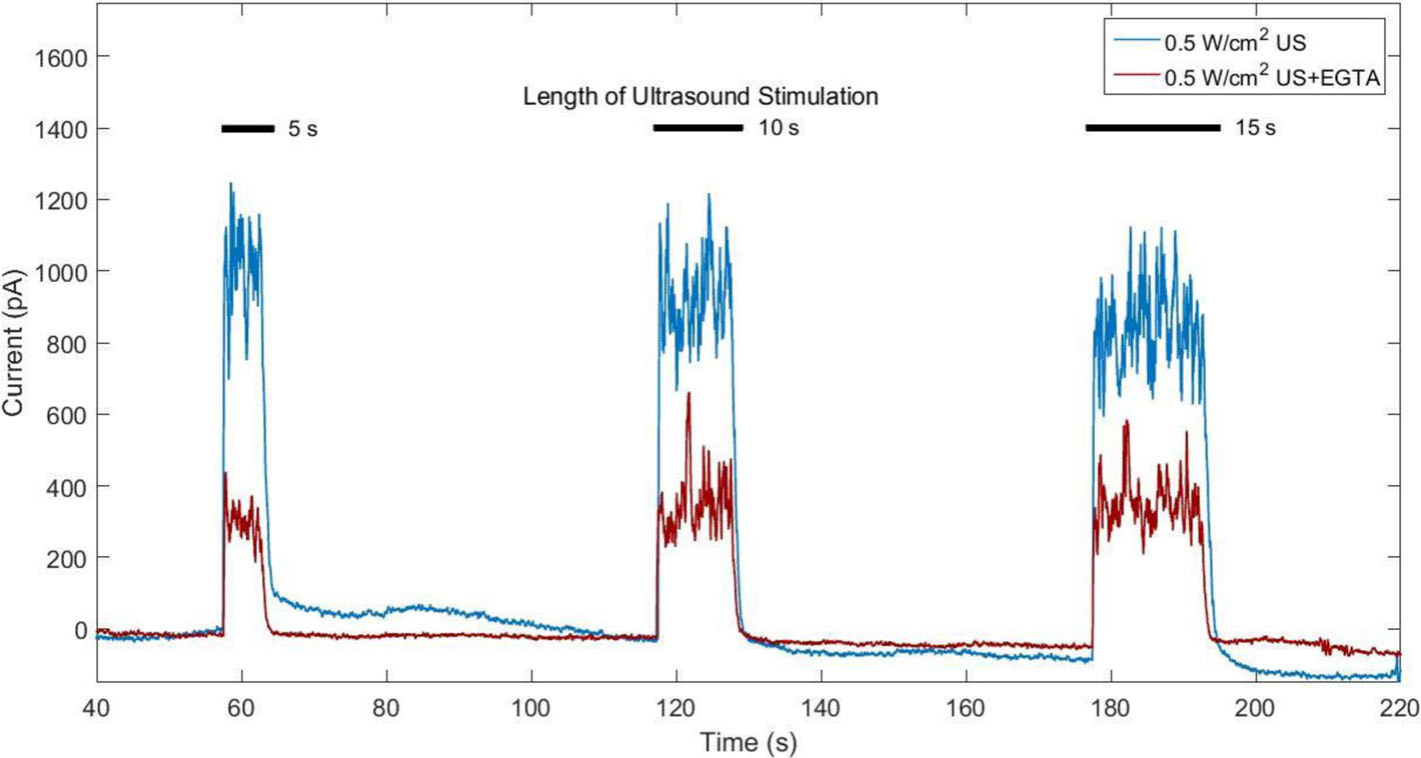
Amperometric detection of dopamine release from 800 kHz, 0.5 W/cm^2^ ultrasound (US)-stimulated beta cells with (blue curve) and without added EGTA (red curve)

The role of Ca^2+^ in ultrasound-stimulated secretions was explored by performing amperometry and ELISA studies in EGTA-containing and EGTA-free samples. An example of amperometric currents detected in response to ultrasound stimulation in EGTA-supplemented KBS (*n* = 6) as compared to KBS with no EGTA (n = 6) is shown in Fig. 7. Five, ten and fifteen-second ultrasound pulses at an intensity of 0.5 W/cm^2^ were applied at *t* = 1 min, *t* = 2 min and *t* = 3 min respectively. Interestingly, when EGTA was added to the extracellular medium as to chelate free extracellular Ca^2+^, peak amplitudes resulting from ultrasound exposure were significantly lower than those detected from samples without Ca^2+^ chelation as shown in Fig. 7 (*n* = 6, *p* < 0.05). Table 4 contains the total charge (mean ± STD) measured after each ultrasound stimulating pulse at intensity of 0.5 W/cm^2^ from beta cells in KBS with and without the presence of EGTA, showing that ultrasound-stimulated secretory events occur in a Ca^2+^-dependent manner.

**Table 4:** Total charge of amperometric peaks in 800 kHz, 0.5 W/cm^2^ ultrasound-treated cells with and without EGTA (*n* = 6, mean ± STD)

			Total charge (pAs)		
Frequency (kHz)	Intensity (W/cm^2^)	EGTA?	1 min (5 s)	2 min (10 s)	3 min (15 s)
800	0.5	NO	4250 ± 911	8212 ± 2255	10,294 ± 2711
800	0.5	YES	1508 ± 611	3162 ± 1666	4246 ± 1976

Our measurements showed that peak amplitudes measured in the presence of EGTA were approximately 64% lower than those measured without EGTA (Table 4, *n* = 6, *p* < 0.05).

**Fig. 8:**
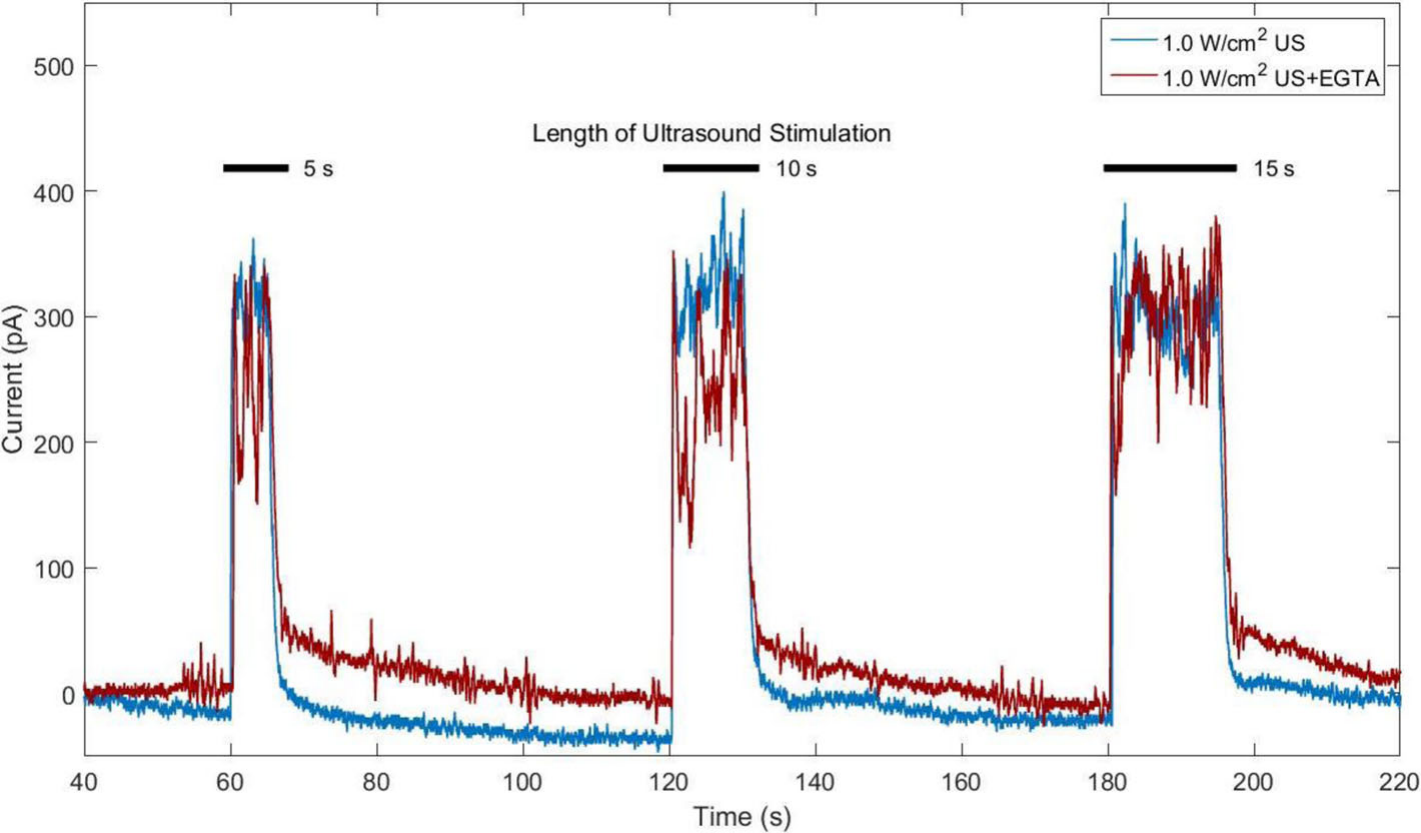
Amperometric detection of dopamine release from 800 kHz, 1 W/cm^2^ ultrasound (US)-stimulated beta cells with (blue curve) and without added EGTA (red curve)

An example of amperometric detection of dopamine release in response to 800 kHz ultrasound stimulation at intensity of 1 W/cm^2^ in EGTA-supplemented KBS as compared to KBS with no EGTA is shown in Fig. 8. Five, ten and fifteen-second ultrasound pulses were applied at *t* = 1 min, *t* = 2 min and *t* = 3 min, respectively. When applying ultrasound at an intensity of 1 W/cm^2^, the peaks detected in the ultrasound-treated group with no EGTA appeared to be higher on average than those detected from the EGTA-containing group (see Fig. 8). However, the amplitude difference between both groups was not as evident as it was when applying ultrasound at intensity of 0.5 W/cm^2^ (see Fig. 7). Table 5 contains the total charge (mean ± STD) measured after each ultrasound stimulating pulse from beta cells in KBS with and without the presence of EGTA. No statistical significance was achieved between measurements of total charge detected from EGTA-containing and EGTA-free ultrasound-treated groups at intensity of 1 W/cm^2^ (Table 5 **,** *n* = 6, *p* > 0.05).

**Table 5:** Total charge of amperometric peaks in 800 kHz, 1 W/cm^2^ ultrasound-treated cells with and without EGTA (*n* = 6, mean ± STD)

			Total charge (pAs)		
Frequency (kHz)	Intensity (W/cm^2^)	EGTA?	1 min (5 s)	2 min (10 s)	3 min (15 s)
800	1	NO	2334 ± 612	3678 ± 757	5434 ± 1634
800	1	YES	1797 ± 553	3509 ± 1188	5063 ± 1065

**Fig. 9:**
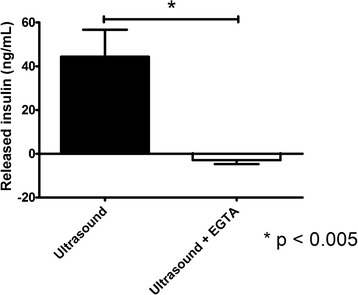
Measurements of insulin release from beta cells exposed to ultrasound with and without Ca^2+^ chelator EGTA (*n* = 6, mean ± SEM)

Figure 9 shows the results of measured insulin release using an insulin ELISA kit from beta cells treated with 800 kHz ultrasound at intensity of 0.5 W/cm^2^ with and without the presence of EGTA. Cells were exposed to 5, 10 and 15 s continuous ultrasound at t = 1 min, t = 2 min and t = 3 min, respectively. While cells exposed to ultrasound with no EGTA released approximately 44 ng/mL of insulin, cells exposed to ultrasound in the presence of EGTA did not release any insulin (*n* = 6, *p* < 0.01). Therefore, ultrasound-induced insulin secretion was completely inhibited by chelating Ca^2+^, thus highlighting the Ca^2+^-dependency of the process. A complete inhibition of insulin release by ultrasound in the presence of EGTA indicates a calcium influx as the major mechanism of ultrasound-stimulated insulin release.

## Discussion

In this study, we analyzed if application of therapeutic levels of ultrasound is capable of safely and effectively stimulating secretions from pancreatic beta cells in physiological, Ca^2+^ dependent manner. Our cell viability studies showed that ultrasound treatment had no detrimental effect on beta cell survival. Cell viability measured immediately after treatment, as measured with trypan blue dye exclusion test, exhibited comparably high percent viable cells as that measured in the sham group. Likewise, results of cell metabolic activity 24 h after treatment, as revealed by MTT cytotoxicity assay, showed no significant difference between ultrasound-treated and sham-treated cells. Results of cell counting performed immediately after the end of treatment showed that there was a minor 9% cell detachment in dishes treated with 800 kHz ultrasound at intensity of 0.5 W/cm^2^ as compared to non-treated dishes. Cells treated with 800 kHz ultrasound at intensity of 1 W/cm^2^ exhibited somewhat levels (> 75%) of cell detachment which prevented us from properly assessing their viability 24 h after treatment. However, we have previously shown that ultrasound exposure for a duration of 5 min using these parameters did not cause significant effect on cell viability as compared to sham-treated cells [24]. Thus, our results demonstrate that ultrasound-induced insulin release is achieved in a safe and physiologically relevant manner.

Carbon fiber amperometry results showed detection of amperometric peaks almost instantly after the start of the ultrasound treatment followed by an immediate return to baseline after turning off the ultrasound. Prolonged stimulations elicited proportionally longer responses as 5, 10 and 15 s ultrasound applications resulted in detected peaks lasting just as long. As expected, negligible responses were detected in non-loaded cells as compared to dopamine-loaded cells treated with the same ultrasound parameters. In fact, the amplitude of the peaks detected in ultrasound-treated cells was about 70 times higher than the amplitude of the current detected from the treatment of unloaded cells. Studies performed at lower ultrasound intensity of 0.1 W/cm^2^ resulted in little to no detected response from treated cells as compared to cells treated at higher intensity of 0.5 W/cm^2^, thus revealing a potential intensity threshold for effective beta stimulation with ultrasound. Our results are comparable to those reported by Bokvist et al. who detected secretory events through electrical membrane depolarization of patched-clamped mouse pancreatic beta cells loaded with serotonin [45]. In this study, the authors measured multiple superimposed amperometric peaks from a single cell representing detection of numerous exocytotic events. Furthermore, the duration of their response appeared to match the duration of the stimulus suggesting that secretion lasts for as long as the cell was depolarized. Our results are consistent with this study and a hypothesis that ultrasound-stimulated secretion is mediated by membrane depolarization and potential Ca^2+^ influx. In support of this idea, our studies demonstrate that chelation of extracellular calcium by EGTA completely inhibited ultrasound-stimulated insulin secretion. Furthermore, the results of our study show that insulin release from beta cells could be temporally controlled by careful selection of ultrasound parameters such as intensity and exposure duration.

Our ELISA studies showed that ultrasound stimulation of beta cells released increasing amounts of insulin as the duration of the exposure was increased from 15 s to 5 min. Thus, amperometry and biochemical studies independently confirmed that the duration and efficacy of insulin and dopamine release from beta-cells can be temporally controlled by the ultrasound-evoked mechanical stimulation of cells. It is worth noting that released quantities of insulin as a result of ultrasound exposure were less than 10% of the total insulin content in these cells which we estimated to be in the range of 1.05 μg to 1.2 μg for a cell count of 700,000 to 800,000 cells [24]. This is an important and biologically relevant finding as excessive insulin release may lead to potently dangerous hypoglycemic effect.

As discussed above, ultrasound applied to cells in the presence of Ca^2+^ chelator EGTA resulted in significant inhibition of the dopamine release as shown by evident decrease in the amperometric peak amplitude (approximately 60% on average). These results suggested that ultrasound-stimulated secretion is at least partially mediated by intracellular Ca^2+^-dynamics. However, peaks were still detected even when the Ca^2+^ in the extracellular space was chelated using EGTA, thus potentially revealing involvement of calcium release from intracellular stores (i.e, mitochondria and/or ER release) or an additional non-Ca^2+^-dependent mechanism of action for ultrasound-stimulated secretory response. Considering that addition of 10 mM EGTA completely inhibited ultrasound-induced insulin release as measured with ELISA, the amperometric peaks detected in the presence of EGTA might be the result of selective release of the small dopamine molecules through “kiss-and-run” exocytosis [46–48].

A Ca^2+^-dependent mechanism mediating ultrasound-stimulated insulin release would involve a pathway resembling that in glucose-stimulated insulin secretion: transient membrane permeabilization to allow ion exchange between the extracellular space and the cell’s cytosol. One possible mechanism would involve direct membrane permeabilization to Ca^2+^ thus triggering the release of secretory vesicle content through exocytosis. Another indirect mechanism would involve transient and non-selective membrane permeabilization allowing cation fluxes (e.g. Na^+^, K^+^ and Ca2^+^) across the cell membrane driven by their standing electro-chemical gradients, which would depolarize the membrane, activate voltage-gated Ca^2+^ channels and stimulate Ca^2+^ influx into the cell that would consequently trigger vesicle exocytosis.

Transient cell membrane permeabilization is an ultrasound-induced bioeffect that has been largely studied in applications such as enhanced drug delivery or gene therapy [49–52]. This process is believed to be primarily mediated by acoustic cavitation (both stable and inertial), a mechanism shown capable of creating resealable pores on the membrane of the cell and allowing the transfer of ions and molecules of different sizes across it [25, 53, 54]. Therefore, it is possible that pores created through cavitation would allow the flow of ions responsible for membrane depolarization (including Ca^2+^) down their respective concentration gradients and stimulate hormone secretion through one of the aforementioned processes [25]. Ca^2+^-dependent hormone secretion stimulated with ultrasound could also be mediated by mechanotransduction, or the stimulation of mechanoreceptors located on the membrane of the beta cell. Stretch-activated cation channels (SACC) sensitive to mechanical stretching of the plasma membrane and volume-regulated anion channels (VRAC) sensitive to cell volume changes are believed to be directly involved in the process of glucose-stimulated insulin secretion [55, 56]. Therefore, activation of SACC and VRAC resulting from physical and subcellular perturbations of the beta cell structure in response to ultrasound pressure could result in intramembrane ion exchange, membrane depolarization, activation of voltage-gated Ca^2+^ channels and subsequent insulin secretion. Pancreatic beta cells are also known to express ion and Ca^2+^-permeable transient receptor potential (TRP) cation channels which have been shown to be sensitive to mechanical stresses and to have a regulatory role in insulin secretion [57, 58]. In particular, one study showed that mechanically stimulating beta cells induced not only Ca^2+^ influx into the stimulated cell, but also generated Ca^2+^ waves that spread to neighboring cells through connexin43 gap junctions and were mediated by autocrine or paracrine activity of secreted ATP acting on P_2U_ purinergic receptors [59].

In other studies, low-intensity ultrasound was reported to cause morphological changes to neuronal cells, a process that the authors believe could have implications in neuronal cell growth and other downstream cellular processes mediated by the cytoskeleton of the cell [60, 61]. The cells later recovered their pre-stimulation shape and size within 30 min after the end of treatment. As such, temporary changes in cell morphology and cytoskeletal disruptions resulting from ultrasound exposure could stimulate machanosensitive membrane proteins (VRAC or SAC), depolarizing the membrane to levels necessary to open Ca^2+^ channels and consequently stimulate insulin secretion. The process known as “intramembrane cavitation” could also be involved in the process of ultrasound-induced insulin release through mechanoreceptor stimulation pathways. The authors suggesting this process believe that the cell membrane is capable of transforming acoustic pressure waves into intracellular deformations [62]. Such cyclic deformations could stimulate cycles of stretch and release in the cell membrane and the cytoskeleton, which in turn could stimulate mechanosensitive proteins, increase membrane permeability and depolarize the cell’s membrane. Other known ultrasound bioeffect that could cause mechanical stresses as to stimulate mechanosensitive channels is acoustic radiation force [63].

Various studies have demonstrated that ionic mechanisms other than the inhibition of K_ATP_ channels might participate in insulin secretion caused by higher glucose concentrations. Among these is beta cell swelling [56, 64, 65], a process shown capable of stimulating insulin secretion and believed to be independent of Ca^2+^ influx. Beta cell swelling is believed to be caused by increased cell metabolism in response to high glucose concentrations, leading to increased intracellular concentrations of lactate [66], Na^+^ and Cl^−^ [67], resulting in intracellular hyperosmolarity and ultimately, insulin secretion. A mechanism by which ultrasound stimulation could induce beta cell swelling would consist of ion accumulation (e.g. Na^+^, Cl^−^) in the cytosol following ultrasound-generated transient membrane permeabilization, thus inducing cell swelling as a result of cellular hyperosmolarity. However, the exact mechanism by which beta cell swelling induces insulin secretion remains unknown [68]. A previous study showed that hormone secretion was not inhibited in hypertonic, beta cell swelling conditions when gadolinium, an inhibitor of mechanoreceptors, was present in the medium [68]. Another study showed that disruption of microtubule and microfilament function had no effect on hyposmolarity-induced secretion [68, 69]. Nonetheless, the process of insulin secretion as stimulated by beta cell swelling was shown to be independent of Ca^2+^ influx [68]. Interestingly, a study showed that Ca^2+^ influx mediated by SACC was able to switch the mode of exocytosis from Ca^2+^-independent “kiss and run” exocytosis to full fusion exocytosis [70]. Therefore, amperometric peaks detected from ultrasound-treated samples in the presence of EGTA might be the result of “kiss-and-run” exocytosis resulting from beta cell swelling.

Although the average amperometry peak amplitude of each response was higher in the dishes that did not contain EGTA when ultrasound was applied at 1 W/cm^2^, no statistical significance was achieved as compared to dishes with EGTA. It is possible that, at higher ultrasound intensities, an alternate mechanism (e.g. beta cell swelling) dominates over the concurrent Ca^2+^-mediated insulin and dopamine release. It is also possible that peaks detected in samples containing EGTA might be the result of dopamine released from the cell’s cytosol following transient membrane permeabilization through one of the mechanisms that were previously discussed. Dopamine is a small molecule with low molecular weight of 153.18 Da. Though incubating beta cells with media supplemented with dopamine and L-DOPA loads and localizes these neurotransmitters in secretory vesicles along with insulin, it is possible that some residual dopamine remains in the cytosol. As previously shown, ultrasound is capable of creating re-sealable pores through stable and inertial cavitation [53, 54]. Studies have reported that, depending on the chosen ultrasound parameters, pore sizes can vary as to allow the passage of a wide range of molecule sizes of up to 155 kDa [54, 71]. Transiently permeating the cell membrane through ultrasound application could allow residual dopamine in the cytosol or dopamine contained in secretory vesicles docked to the cell membrane, to exit the cell and oxidize the carbon fiber electrode. Therefore, amperometric peaks generated by dopamine released from secretory vesicles through Ca^2+^-dependent exocytosis might have been masked by larger amounts of dopamine exiting the cytosol through large pores created by application of higher ultrasound intensity. However, based on our results, this mechanism would require pores to reseal almost instantly after the end of ultrasound treatment, mimicking the temporal profile of the detected response. Studies have reported that ultrasound-created pores usually have a lifetime ranging from milliseconds to several seconds [72]; with some studies reporting longer resealing times of at least 1 min [54]. Therefore, we would expect to continue detecting amperometric peaks for some time after the end of ultrasound stimulation. In addition, the high polarity and poor lipid solubility of dopamine would significantly affect its ability to cross the phospholipid bilayer in a passive manner.

## Conclusions

The results of this study demonstrated that 800 kHz ultrasound at intensity of 0.5 W/cm^2^ is capable of safely stimulating insulin secretion of up to 10% of its pancreatic beta cell content within minutes of stimulation, thus mirroring the rate and amount of glucose-evoked insulin release from native islet beta cells. Furthermore, we showed that stimulated secretions lasted as long as the durations of the ultrasound stimulus, thus suggesting that tight control of the amount of released insulin may be achieved by careful selection of ultrasound parameters such as intensity and exposure duration. In addition, application of ultrasound at lower intensities of 0.1 W/cm^2^ yielded much lower responses (approximately 87% lower), indicating the possibility of a pressure threshold that must be achieved before secretion takes place. Finally, the process of ultrasound-stimulated secretory events was shown to be Ca^2+^-dependent much like the action of other known insulin secretagogues. Future studies will include application of the ultrasound parameters used in this study to more physiologically relevant models such as pancreatic islets and diabetic rodent animal models.
